# Practice Recommendations for Genetic Testing of Ataxias

**DOI:** 10.1002/acn3.70171

**Published:** 2025-08-29

**Authors:** Sharan R. Srinivasan, Amy D. Mook, Michelle Rochman, Jin Yun Helen Chen, Weiyi Mu, George R. Wilmot, Liana S. Rosenthal, Wendy R. Uhlmann

**Affiliations:** ^1^ Department of Neurology University of Michigan Ann Arbor Michigan USA; ^2^ Department of Neurology Thomas Jefferson University Philadelphia Pennsylvania USA; ^3^ Department of Neurology Mass General Hospital Boston Massachusetts USA; ^4^ Department of Neurology Johns Hopkins University Baltimore Maryland USA; ^5^ Department of Neurology Emory University Atlanta Georgia USA; ^6^ Division of Genetic Medicine, Department of Internal Medicine University of Michigan Ann Arbor Michigan USA; ^7^ Department of Human Genetics University of Michigan Ann Arbor Michigan USA

**Keywords:** ataxia, genetic counseling, genetic testing, genomics, hereditary

## Abstract

**Objective:**

Over the past decade, significant advances in genetic testing for ataxia have improved diagnostic accuracy, informed clinical trial eligibility, guided treatment decisions, and enabled cascade testing of at‐risk relatives. While guidance exists for other neurogenetic conditions, there are no standardized guidelines on genetic counseling and testing for individuals with unexplained ataxia.

**Methods:**

We conducted a comprehensive literature review on genetic counseling and testing in ataxia, identifying 7362 articles. After removing 2971 duplicates, 4391 articles were screened by two authors using the Evaluation of Genomic Applications in Practice and Prevention (EGAPP) framework. In areas lacking clear published evidence, we convened a multidisciplinary expert panel with clinical and genetic expertise in ataxia. Following conflict resolution and additional filtering, 68 articles were included in our guidance development.

**Results:**

Based on this evidence and expert consensus, we developed 20 recommendations addressing indications for genetic testing in hereditary ataxia, components of pre‐ and post‐test counseling, testing options, insurance considerations, interpretation of test results, and appropriate referral to genetic counseling services. Major themes include the importance of formal genetic counseling and suggesting whole genome sequencing as first‐line testing, with an emphasis on detecting repeat expansions.

**Conclusion:**

These evidence‐based, consensus‐driven recommendations aim to support clinicians in evaluating patients with unexplained ataxia in order to provide timely evaluation and care, both for patients and their at‐risk relatives.

## 1 Introduction

Cerebellar ataxias comprise a group of neurological disorders characterized by gait instability, dysarthria, and appendicular dysfunction [1, 2]. These disorders may present with isolated ataxia or with additional neurological symptoms, such as parkinsonism, dystonia, tremors, and cognitive impairment. When sporadic, cerebellar ataxias may arise from paraneoplastic or other autoimmune diseases, vitamin deficiencies, medication side effects, and other toxins [3]. There are also age‐based neurodegenerative disorders presenting with ataxia, for which causes are less clear. Workup generally will include imaging (e.g., MRI), serological and CSF studies [4]. A large subset of ataxias, though, are hereditary in nature, for which genetic testing is a key part of the workup.

Hereditary ataxias are a group of distinct disorders, each with its own genetic basis. There are over 60 spinocerebellar ataxias with different inheritance patterns [5, 6, 7, 8] and new causative genes continue to be identified. Some genetic ataxias can mimic other neurodegenerative disorders [9, 10, 11, 12] and must be considered as part of the diagnostic workup. Genetic testing options for ataxia now include single gene sequencing, multigene panels, repeat expansion analysis, and whole genome sequencing. The testing technology required for identification of each genetic ataxia may differ depending on the genetic variant(s) in question (e.g., sequence variant vs. repeat expansion).

Genetic testing plays a key role in confirming diagnoses, guiding management, enabling clinical trial access, and informing family risk. In decision‐making about genetic testing, there are several patient‐specific considerations including test and lab selection, insurance implications, coverage of testing cost, use for family planning, and personal perceptions of testing utility. Ideally, genetic counseling would be provided by a board‐certified genetic counselor. However, in the absence of this resource, information can be provided to individuals by a neurologist with dedicated neurogenetics training or a geneticist. Additional information about access to genetic counseling services and insurance billing is described in Appendices A and B.

There are limited studies with guidance for providing genetic counseling and testing for ataxia. Most available information pertains to molecular testing methods or represents singular forms of ataxia [13, 14]. In searching for guidance on genetic counseling and testing in neurogenetic diseases, we looked to two neurodegenerative disorders where guidelines have been well established, Huntington Disease (HD) [15, 16, 17, 18, 19] and amyotrophic lateral sclerosis (ALS) [20]. While HD is solely due to a repeat expansion in the *HTT* gene [21] with autosomal dominant inheritance, ataxia can result from mutations in various genes with diverse inheritance patterns. Similarly, both ALS and ataxia have multiple genetic and non‐genetic causes. For ALS, rapid genetic testing is crucial due to the disease's fast progression and the need for timely referral to clinical trials. Most genetic ataxias, however, progress slowly, with care focused on preventing decline through assistive devices and specialized physical therapy.

Given these differences, there is a need for clear and standardized recommendations on genetic counseling and testing for unexplained ataxia. The evaluation of ataxia is complex, and the selection of appropriate genetic testing is essential. Early diagnosis can improve the quality of life for patients and at‐risk family members. Building on existing HD and ALS guidelines, along with our literature review and clinical experience, we offer recommendations for genetic counseling and testing of ataxias.

## 2 Methods

### 2.1 Author Group

The author group was led by a physician scientist (S.R.S.) and included five genetic counselors (A.D.M., M.R., J.Y.H.C., W.M., and W.R.U.) along with two additional physician scientists/neurologists (G.R.W. and L.S.R.). This group includes a wealth of experience working with individuals with ataxia and their families and/or the application of genetic testing and counseling for neurodegenerative conditions. Additional contributions to this work include input from the Clinical Research Consortium for the Study of Cerebellar Ataxia (CRC‐SCA) and the National Ataxia Foundation.

### 2.2 Clinical Scope

These recommendations apply to four groups: (1) symptomatic individuals with ataxia (diagnostic), (2) asymptomatic individuals with a family history (predictive), (3) those pregnant or planning pregnancy with a personal/family history (reproductive), and (4) children with ataxia (pediatric). Details for each group are in Table 1. While focused on cases where ataxia is the primary indication, we acknowledge that ataxia‐related variants may also be found during testing for other conditions (e.g., parkinsonism, chorea, dystonia, spastic paraparesis); in such cases, recommendations may still apply but should be adapted accordingly. Institutional practices may affect implementation. As all authors practice in the United States, these guidelines are U.S.‐specific, though many may be relevant internationally depending on local healthcare systems and laws.

**TABLE 1:** Genetic testing scenarios and definitions.

Type of testing	Definition
Diagnostic	Performed for an ataxic individual to either establish or confirm a suspected diagnosis. Testing may be requested for a variety of reasons, including the need to determine an explanation for symptoms, a desire to definitively name one's disease, and inform healthcare and life decisions. *See recommendations #1–4, 6, 9–20*
Predictive	Performed for an asymptomatic individual with a family history of ataxia. This may be requested for a variety of reasons, including future planning for marriage, reproduction, career, home, insurance, finances, travel or simply a need to resolve uncertainty. Although there are currently no direct medical benefits from predictive testing, there are lifestyle modifications, such as cardiovascular exercise, which is shown in animal models to be protective against disease onset and progression [22, 23, 24]. *See recommendations #5–6, 9–17*
Reproductive	Performed when an individual with a known genetic ataxia or at risk of a genetic ataxia seeks genetic testing either prior to (preconception) or during a pregnancy (prenatal). This may be requested to make decisions about testing a pregnancy or reproductive options, such as IVF or donor gametes. If a pregnancy is tested, results may be used to decide whether or not to continue a pregnancy. *See recommendations #6–7, 9–17*
Pediatric	Performed when a child (individual under age 18) is symptomatic for an ataxia syndrome to either establish or confirm a suspected diagnosis. Testing may be requested to determine an explanation for symptoms and inform healthcare decisions. *See recommendations #6, 8–18*

### 2.3 Literature Review

The following databases were searched on November 6, 2024, without any time period restrictions, to identify relevant articles, trials, or meeting abstracts describing genetic counseling and testing in ataxia: PubMed.gov, Elsevier Embase (including Embase Classic), and Elsevier Scopus. No limits were applied to the search. A set of sentinel articles from the ataxia literature, alongside the published ALS [20] and HD [19] guidelines, was identified before the search process and was used to generate search terms and test the effectiveness of the strategies in each database. Reference tracking was performed on highly relevant articles. Original search strategies were developed in PubMed and translated as appropriate to the other databases using the Systematic Review Accelerator Polyglot tool [25]. Citations were deduplicated using Covidence's deduplication tool. The complete search strategies are available in Supporting information.

To guide the literature review, the following clinical questions were identified:

1. *Neurological Evaluation*: When should persons with a personal or family history of ataxia undergo neurological evaluation and at what point should genetic testing be considered?
2. *Genetic Counseling/Genetic Counselor Involvement*: What information should be provided to persons with a personal or family history of ataxia before and after testing?
3. *Timing*: At what time or in what scenarios should genetic testing be offered to persons with a personal or family history of ataxia?
4. *Informed Consent*: What information should be provided to persons with a personal or family history of ataxia to obtain informed consent for genetic testing?
5. *Test Selection/Laboratory Methods*: What test methodologies and approaches should be considered when selecting genetic testing for persons with a personal or family history of ataxia?

The following *exclusion criteria* were developed prior to and applied during our literature review. All articles that did not meet any exclusion criteria were then included in the next phase of review.

*Title/Abstract review*

- Language not in English and no transcription readily available
- Not applicable to clinical questions
- No full text available (e.g., abstract was a conference submission or proceeding)

*Full text review*

- As above with title/abstract review
- Article is either a case report or editorial/letter to editor
- Ataxia is < 25% of the patient population; research is not specific to ataxia.
- Article is prior to 2015 (for questions regarding diagnostic yield and specific testing)
- Article is not peer reviewed
- Article has < 5 years of longitudinal data (when assessing a single center results or diagnostic yield)

**FIGURE 1:**
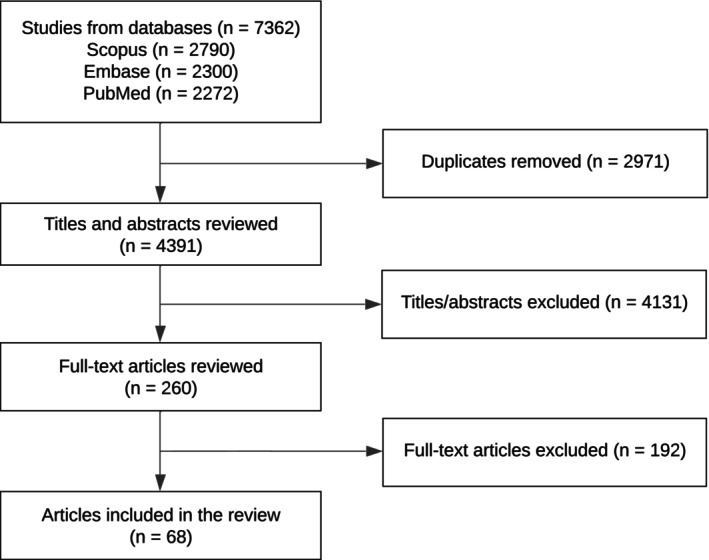
PRISMA flow diagram of the identified studied (68 articles were included in the review).

A total of 7362 relevant articles were identified in this search. 2971 citations were duplicates and removed. S.R.S. and A.D.M. performed title and abstract review of the remaining 4391 citations based on above “Title/Abstract Review” criteria, consideration of the above clinical questions, and resolution of any nonunanimous decisions. Full‐text review was conducted on 260 articles. Ultimately, 68 articles were selected for inclusion based on “Full Text Review” criteria above. This process is outlined in a PRISMA diagram in Figure 1. Relevant information and data were pulled from these citations.

### 2.4 Initial Practice Recommendation Development

21 initial practice recommendations were developed based on the information obtained from the literature review. These were each categorized in relation to the clinical question addressed: eight recommendations regarding neurological evaluation, ten recommendations regarding genetic counseling, and three recommendations regarding test selection and laboratory reporting/methods. Some of the genetic counseling recommendations have content relevant to timing and informed consent. These practice recommendations were graded based on the adapted Evaluation of Genomic Applications in Practice and Prevention (EGAPP) used in the development of the guidelines for ALS genetic testing and counseling [20].

### 2.5 Expert Opinion Panel

The expert opinion panel was established via invitation based on discipline or expertise. Experts included academic neurologists, community neurologists, genetic counselors, and representatives from the National Ataxia Foundation.

**FIGURE 2:**
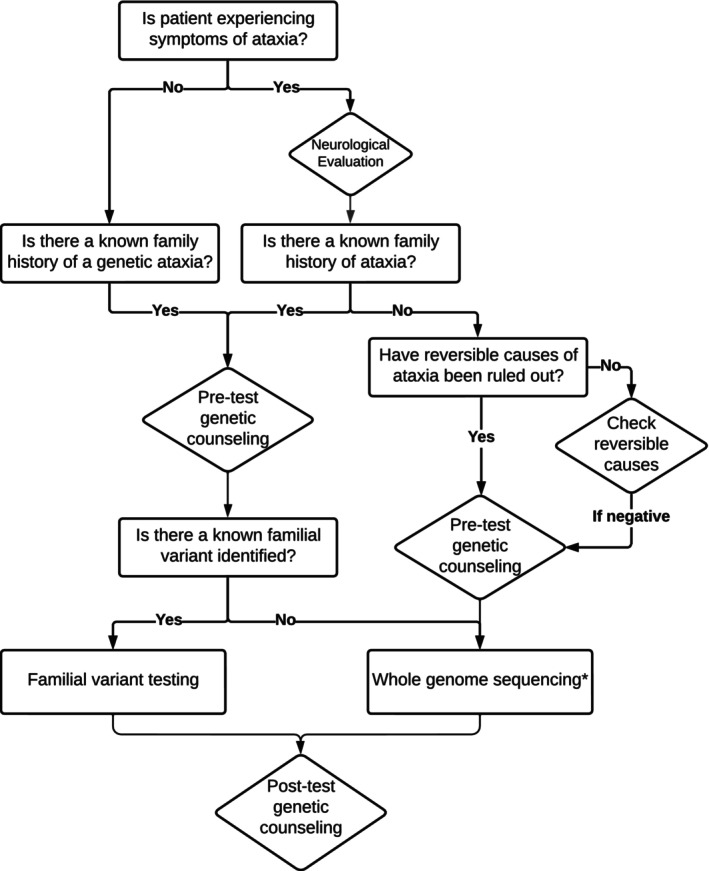
Proposed workflow for evaluation of hereditary ataxia. Genetic counseling may be conducted by a genetic counselor, neurologist with dedicated neurogenetics training, or geneticist based on suspected indication and level of comfort in test selection/interpretation. *Whole genome sequencing (WGS) must include technology capable of reliably detecting repeat expansions. See *Recommendation #18* for more details, including alternative testing options such as a dedicated repeat expansion panel.

### 2.6 Modified Delphi Consensus

A modified Delphi consensus approach was used to finalize the practice recommendations using a Research Electronic Data Capture (RED‐Cap) survey. This survey link was distributed via email to all individuals that agreed to participate as a member of the expert opinion panel. Members were given draft recommendations and asked to vote “yes” or “no” with comments. Consensus was established prior to voting as ≥ 80%.

Following the first round of voting, 14 of 21 original recommendations achieved consensus. After reviewing feedback, one recommendation was consolidated into another. Six recommendations then underwent a second round of voting, with all achieving consensus.

## 3 Results

A final 20 recommendations are thus presented below and in Table S1 with the specific article(s) cited as evidence for each recommendation. For ease of reference, the recommendations have been categorized into three sections: (1) Clinical Decision Making, (2) Genetic Counseling, and (3) Genetic Testing and Laboratory Methodology. An overall workflow is presented in Figure 2. Additional information with regard to genetic counseling, including access to services, is available in Appendices A–C.

### 3.1 Section 1: Practice Recommendations for Clinical Decision Making

*Recommendation #1: All symptomatic persons should undergo a comprehensive evaluation by a neurologist prior to initiating genetic testing*.

Strength: This recommendation is supported by at least one study of level 2 evidence.

Testing scenarios: Diagnostic.

All symptomatic patients should undergo a comprehensive evaluation by a neurologist to define the presence and extent of ataxic symptoms and ensure there are no reversible or non‐genetic etiologies [26, 27, 28]. For those diagnoses where genetic etiology is unclear, phenomenology and family history of other neurological disorders may guide specific testing.

*Recommendation #2: For symptomatic persons* *without a known* *family history, non‐genetic causes of ataxia should first be ruled out prior to genetic testing, unless clinical suspicion is high for a particular syndrome*.

Strength: This recommendation is supported by at least one study of level 2 evidence.

Testing scenarios: Diagnostic.

For patients without a positive family history, reversible and/or sporadic causes of ataxia should first be evaluated. This may include imaging, serological, or even CSF studies as deemed appropriate by the neurologist. Following negative reversible testing, consideration of genetic causes is then appropriate (see *Recommendation #18*). However, there are certain patient presentations that, even without knowledge of family history, may raise clinical suspicion high enough to the point of directly proceeding with genetic testing (e.g., early symptom onset).

*Recommendation #3: For persons with a known family history of ataxia and a neurological evaluation consistent with that syndrome, genetic testing should be the first diagnostic test ordered*.

Strength: This recommendation is supported by at least one study of level 2 evidence.

Testing scenarios: Diagnostic.

For patients with a positive family history and consistent clinical symptoms, the same genetic cause of ataxia is likely. Therefore, it is appropriate to proceed directly to genetic testing, either for a known familial variant if a genetic cause has been established in the family or via comprehensive genetic testing (see *Recommendation #18*). Formal genetic counseling may be offered but should not delay testing. If the patient has discordant clinical symptoms or the clinical presentation is atypical for the genetic ataxia in question, then additional workup such as imaging or serological studies may be appropriate prior to genetic testing.

*Recommendation #4: Ordering genetic testing should be expedited if there is high clinical suspicion of a genetic ataxia with an FDA‐approved treatment*.

Strength: This recommendation is supported by at least one study of level 2 evidence.

Testing scenarios: Diagnostic.

At time of publication, the only FDA approved therapies for *any* ataxia are for treatment of Friedreich's Ataxia (FA) [29, 30, 31]. Given that an accurate diagnosis would change clinical management, there is rationale to expedite testing for FA if clinical suspicion is high. Similarly, as emerging therapies for other ataxias become approved, testing for those conditions would become more urgently indicated.

*Recommendation #5: All asymptomatic persons with a known family history of a genetic ataxia should be offered genetic counseling*.

Strength: This recommendation is supported by at least one study of level 3 evidence.

Testing scenarios: Predictive.

Optimally, genetic counseling should be performed by a genetic counselor. In the absence of this resource, a neurologist with dedicated neurogenetics training or a geneticist is preferable. This does not obligate the person to proceed with testing but is recommended to better understand the risk of the familial condition and the potential impact of results should they proceed with testing. This guidance is in keeping with recommendations for predictive genetic testing for other neurogenetic conditions [20, 32, 33, 34, 35].

*Recommendation #6: Psychological status of every patient should be considered. While not a formal requirement prior to genetic testing, referral should be made at the discretion of the ordering provider*.

Strength: This recommendation is supported by at least one study of level 3 evidence.

Testing scenarios: Diagnostic, Predictive, Reproductive, Pediatric.

One distinctive feature in the HD protocol is the recommendation for pre‐test psychological evaluation. Given the neuropsychiatric features in HD, including increased suicidality [36, 37], this was an agreed‐upon measure to ensure patient readiness and safety, especially when receiving a positive result. Most ataxia patients, though there are certainly exceptions, are mainly debilitated from a motor standpoint. Comorbid psychiatric conditions such as depression and anxiety are common [38], and ataxic patients do have an increased risk of suicidal ideation [39]; however, these comorbidities are often not the predominant phenotype. As such, our suggestion is to not formally recommend a psychological or psychiatric evaluation but rather leave it to the discretion of the ordering provider. Patients should be asked about a history of depression, anxiety, and suicidal ideation, and inquiry made as to whether they are seeing a therapist and their sources of support. Consideration should be given to referring the patient to a therapist/psychiatrist as indicated and potentially deferring testing until appropriate mental health care is established [19]. Lastly, it is also important for individuals who decide to have testing to proceed when timing is optimal and ensure appropriate support is in place.

*Recommendation #7: Persons with a known personal or family history of a genetic ataxia who wish to test a pregnancy should be referred to a genetic counselor prior to or early in a pregnancy*.

Strength: This recommendation is supported by at least one study of level 2 evidence.

Testing scenarios: Reproductive.

For individuals with a personal and/or family history of known pathogenic variant(s) for hereditary ataxia, it is possible to do preimplantation genetic testing (PGT) and prenatal testing (chorionic villus sampling or amniocentesis) [40, 41, 42]. Individuals with an autosomal recessive ataxia should be referred with their reproductive partner (if available) to discuss carrier screening for the partner and genetic testing options for a pregnancy if the partner is also a carrier. Timely genetic counseling is critical to determine if testing a pregnancy is an option (depends on whether pathogenic variant has been identified in patient and/or family member) given the time needed to complete genetic testing and the potential time‐sensitive decisions for pregnancy management. Genetic testing of the fetus for hereditary ataxias should not be done if pregnancy management will not be affected [43]. Additional information on reproductive testing can be found in Appendix C.

*Recommendation #8: Genetic testing of minors should only be offered when the minor is determined to be symptomatic by a pediatric neurologist with expertise in ataxia. Testing of asymptomatic minors may be considered if there is an approved treatment in childhood and there is a positive family history with an identified or suspected pathogenic variant(s)*.

Strength: This recommendation is supported by at least one study of level 2 evidence.

Testing scenarios: Pediatric.

If a child is symptomatic, evaluation by a pediatric neurologist with expertise in ataxia is recommended; the scenario becomes similar to adult diagnostic testing as above.

If a child is asymptomatic, then predictive genetic testing for an adult‐onset ataxia should not be performed until age 18 or older in accordance with standard genetic testing guidelines [44, 45, 46]. In rare cases, a request for testing by a mature minor close to the age of majority may be considered, and the ability to *assent* may be determined on a case‐by‐case basis. The exception to this non‐testing of minors recommendation would be where positive test results would change clinical management in childhood/early adulthood, for example, where a therapeutic option exists. It is especially important to note the potential implications of testing positive on insurance coverage since minors generally are not aware of these long‐term implications.

#### 3.1.1 Section 2: Practice Recommendations for Genetic Counseling

*Recommendation #9: All individuals with or at risk for a suspected genetic ataxia should be offered genetic counseling*.

Strength: This recommendation is supported by at least one study of level 3 evidence.

Testing scenarios: Diagnostic, Predictive, Reproductive, Pediatric.

As above, counseling can be provided by either a genetic counselor, neurologist, or geneticist. However, if there are other neurological conditions in the family, the patient may benefit from a genetics referral prior to genetic testing to determine specific tests to order or post‐testing to determine if additional testing is indicated given the results.

Of note, the 21st Century CURES Act allows patients immediate access to their medical data, including genetic testing results being available directly to patients, even prior to provider receipt (Public Law 114–255). See Tables 2 and 3 for additional key points to be discussed with a patient as part of genetic counseling.

**TABLE 2:** Key points to include in pre‐test genetic counseling.

Pre‐test counseling
Collect medical history, including patient‐perceived symptoms and time course of ataxia
Collect a three‐generation family history and ages of onset in affected individuals
Provide personal risk assessment based on inheritance pattern(s) of suspected ataxia/neurological condition(s).
Test selection ○ Determine if ordering proband only versus duo, trio, or quad testing with additional family members ○ If relevant, review additional information that the patient may opt in or out of receiving (e.g., secondary or incidental findings)
Review types of tests and results ○ Repeat expansion analysis: positive, negative, intermediate ○ Sequencing and deletion/duplication analysis: positive, negative, VUS
Discuss benefits, risks, and limitations of genetic testing, and personal utility ○ Review cost of genetic testing, billing options and whether clinic or patient will need to contact lab/insurer to determine if testing will be fully, partially, or not at all covered ○ Review possible implications of genetic test results for insurance coverage (e.g., life)
Timing of genetic testing ○ Discuss if timing is optimal if requested close to holidays or major lifecycle events
For patients who do not undergo testing, more general resources on ataxia should be provided.
Obtain informed consent, if proceeding with testing
Determine plan for communication of results with consideration of modality and timing with recognition of potential immediate release of results to patients due to the 2021 CURES Act

**TABLE 3:** Key points to include in post‐test genetic counseling.

Post‐test counseling
Patients should be provided with a copy of their results report and appropriate counseling as dictated by their results type (see Table S2) [47, 48, 49, 50, 51, 52]
For a negative or uncertain result, patients should be encouraged to periodically reach out for updates and additional testing recommendations (if any) due to the evolving knowledge of genetic ataxias
For a positive result: ○ Discuss any changes to management, including availability of FDA‐approved therapies or treatment, research opportunities including clinical trials ○ Discuss implications for relatives and recommendations for genetic testing
Provide disease‐specific resources relevant to the patient including support groups. If no specific ataxia is identified, more general resources can be provided

*Recommendation #10: Informed consent must be obtained before genetic testing is ordered*.

Strength: This recommendation is supported by at least one study of level 3 evidence.

Testing scenarios: Diagnostic, Predictive, Reproductive, Pediatric.

As with any medical procedure, informed consent must be obtained prior to clinical genetic testing. This ensures protection for both the individual being tested and the clinical providers. Informed consent, as defined by the Belmont Principle [53], includes three elements: beneficence, respect for persons, and justice. Of note, written consent is required in some states [54]. In the U.S., the National Human Genome Research Institute maintains a genome statute and legislation database which can be used to find out laws in specific states: https://www.genome.gov/about‐genomics/policy‐issues/Genome‐Statute‐Legislation‐Database.

*Recommendation #11: The individual seeking genetic testing must be determined to have the capacity for decision making, unless a medical power of attorney has been appointed*.

Strength: This recommendation is supported by Expert Opinion.

Testing scenarios: Diagnostic, Predictive, Reproductive, Pediatric.

It is critical that the provider ensures that the individual has the *capacity* to make a decision regarding genetic testing. For those patients without capacity, for example, individuals with cognitive limitations, a medical power of attorney acting in the best interest of the patient is required. Central to making an informed decision is the individual/guardian/power of attorney (POA)'s ability to understand the rationale of doing genetic testing and the pros and cons of proceeding, deferring, or declining testing.

*Recommendation #12: All genetic testing must be performed free of coercion*.

Strength: This recommendation is supported by at least one study of level 3 evidence.

Testing scenarios: Diagnostic, Predictive, Reproductive, Pediatric.

The decision to pursue genetic testing must always be an informed, carefully considered, and freely chosen personal decision by the individual (or guardian/POA). It must never be pressured by a partner, family member, healthcare provider, insurance provider, or an employer.

*Recommendation #13: Genetic counseling should include a discussion of the benefits, risks, and limitations of genetic testing*.

Strength: This recommendation is supported by at least one study of level 3 evidence.

Testing scenarios: Diagnostic, Predictive, Reproductive, Pediatric.

It is the responsibility of the healthcare professional to help the individual requesting the test to consider the benefits, the potential risks, and limitations of genetic testing and types of test results (Table S2). Potential benefits include that a patient may become eligible for an FDA‐approved treatment or clinical research if a pathogenic variant is identified. Additionally, a positive genetic test may reduce additional clinical workup and enable the provider to counsel the patient about the symptoms associated with the specific ataxia identified. Potential risks include changes in the individual's perception of self, hypervigilance and anxiety over possible symptoms, changes in familial relationships, and insurance implications (see *Recommendation #14* and Appendix B) [55, 56]. All genetic testing is limited by the currently available technologies and knowledge of genetic drivers of ataxia at the time of testing and results interpretation.

*Recommendation #14: Pre‐test genetic counseling should include a discussion of current legal protections and insurance implications*.

Strength: This recommendation is supported by at least one study of level 2 evidence.

Testing scenarios: Diagnostic, Predictive, Reproductive, Pediatric.

In the U.S., the Genetic Information Nondiscrimination Act (GINA) [57, 58, 59] was passed in 2008 as a federal law to protect patients from genetic discrimination in insurance and employment. Genetic information is defined as genetic test results, family medical history, and genetic services (requested or received). GINA is divided into two sections or Titles:

- Title I: Prohibits discrimination based on genetic information in health coverage. This includes premiums, coverage decisions, or impositions about preexisting condition exclusions.
- Title II: Prohibits discrimination based on genetic information in employment. This includes hiring, firing, salary, job assignments, promotions, layoffs, training, and fringe benefits.

It is crucial to understand and convey to patients that GINA has caveats including that it does not apply to symptomatic individuals, employers with < 15 employees, individuals receiving care through Federal Employee Health Benefits programs, the U.S., military (Tricare), or the Indian Health Service among others. It also does *not* protect against discrimination based on genetic information in the following:

- Life Insurance
- Disability Insurance
- Long‐Term Care Coverage

Information about GINA can be accessed at www.ginahelp.org and https://www.genome.gov/about‐genomics/policy‐issues/Genetic‐Discrimination#gina. The National Human Genome Research Institute also maintains a database on state laws; some states go beyond the scope of GINA: https://www.genome.gov/about‐genomics/policy‐issues/Genome‐Statute‐Legislation‐Database. Again, more information is available in Appendix B.

*Recommendation #15: Post‐test genetic counseling should be provided to all persons regardless of type of result*.

Strength: This recommendation is supported by at least one study of level 3 evidence.

Testing scenarios: Diagnostic, Predictive, Reproductive, Pediatric.

Given the significant implications of genetic testing results for both the patient and their family members, we recommend that patients be seen for post‐test counseling. To elaborate, a positive result may prompt changes in clinical care, change family planning, and/or prompt cascade testing. A VUS result merits post‐test counseling to determine if VUS resolution is an option and to ensure the patient understands the limitations in interpretation and plans for follow‐up. As negative results may prompt further testing or have diagnostic implications itself, these patients should also receive post‐test counseling. Depending on the result and the patient's understanding, a referral to a genetic counselor, neurologist with neurogenetics training, or geneticist may be indicated. Specific discussion points are detailed in Tables 2, 3, and S2.

*Recommendation #16: For individuals for whom a genetic cause is identified, post‐test counseling should include a discussion of carrier screening or predictive testing for at‐risk family members*.

Strength: This recommendation is supported by at least one study of level 3 evidence.

Testing scenarios: Diagnostic, Predictive, Reproductive, Pediatric.

In this situation, those family members should be referred for genetic counseling (see *Recommendation #5*). Testing of asymptomatic minor siblings at risk is discussed in *Recommendation #8*. Additional information on reproductive testing can be found in Recommendation #7 and Appendix C [60, 61, 62, 63, 64].

*Recommendation #17: Individuals undergoing genetic testing are encouraged to have a support person present, especially at result disclosure*.

Strength: This recommendation is supported by at least one study of level 3 evidence.

Testing scenarios: Diagnostic, Predictive, Reproductive, Pediatric.

Often this is a spouse/partner but may be an adult child or close friend. Generally, another at‐risk individual (e.g., sibling, adult child) is not recommended given potential issues of dealing with their own risks while simultaneously providing support. However, it should also be recognized that the individual may feel that a sibling may be their preferred support because of their shared experiences or specifically may want an adult child(ren) there to hear the information.

#### 3.1.2 Section 3: Practice Recommendations for Genetic Testing and Laboratory Methodology

*Recommendation #18: The initial genetic test ordered should be as comprehensive as possible, include the most ataxia‐relevant genes, and reliably detect pathogenic repeat expansions*.

Strength: This recommendation is partly supported by at least one study of level 1 evidence.

Testing scenarios: Diagnostic, Pediatric.

Novel genetic causes for hereditary ataxia are being discovered with increasing frequency. Currently, the most common genetic ataxias are repeat expansion disorders. Traditionally, this has required dedicated repeat‐primed PCR and sequencing to reliably detect and quantify a repeat number. However, to reduce patient burden, account for newly discovered genes, and maximize currently available technology, *in the ideal case*, initial genetic testing should include *whole genome sequencing (WGS) with the ability to accurately size short tandem repeats*. This may include short read WGS with reflex to long‐range sequencing such as Oxford Nanopore or PACBio, complementary repeat‐primed PCR, or other emerging technologies. This strategy is well documented to increase diagnostic yield [65, 66, 67, 68, 69, 70, 71, 72, 73, 74, 75, 76, 77] and account for novel gene discovery when WGS is reanalyzed. To further increase diagnostic yield, phenotypic information and/or clinical suspicion should be provided at the time of ordering the test. If WGS as a first approach is fiscally impossible or otherwise not feasible, then a dedicated repeat expansion panel with eventual reflex to WGS would be appropriate. We recommend reviewing ACMG policy statements [78, 79] for proper WGS counseling guidance. As in *Recommendation #2*, high clinical suspicion can prompt single gene testing, if appropriate.

*Recommendation #19: Reanalysis of previously negative genomic testing should be considered after a period of at least 2 years or a change in symptomology*.

Strength: This recommendation is supported by at least one study of level 4 evidence.

Testing scenarios: Diagnostic.

Given the newness of genomic tests and limitations with variant interpretation, many genetic testing companies frequently store sequencing data for periodic reanalysis, though policies can vary. If an individual has had whole exome sequencing (WES) or WGS testing in the past without a positive result or if there is onset of new symptoms, we recommend the clinician contact the clinical laboratory that did the testing and request reanalysis if it has been at least 2 years. This includes detection of novel repeats, if applicable.

*Recommendation #20: DNA banking should be offered to all symptomatic individuals for a known or suspected genetic ataxia who either have negative results or decline testing*.

Strength: This recommendation is supported by Expert Opinion.

Testing scenarios: Diagnostic.

DNA banking is the long‐term storage of an individual's DNA. Especially for individuals with a life‐limiting condition who either have had uninformative genetic testing or declined testing, banking DNA facilitates having an affected individual's sample available for future testing as advances in testing are made. Identifying a pathogenic variant in an affected individual enables at‐risk family members to have the option of genetic testing. Generally, the cost is not covered by insurance. Labs that offer DNA banking can be identified through the NIH Genetic Testing Registry (www.ncbi.nlm.nih.gov/gtr/) or an internet search.

## 4 Conclusion

Genetic testing is becoming a vital diagnostic tool for ataxia. To support clinicians, we developed practice recommendations based on literature review, EGAPP grading, a modified Delphi consensus, and clinical experience. These U.S.‐based guidelines draw from related neurological conditions and the expertise of ataxia specialists and genetic counselors. While some aspects may vary internationally, the recommendations offer a practical framework for timely and effective genetic evaluation. It should be noted that genetic testing is a rapidly evolving field. It is therefore possible that novel methodological approaches may emerge that prompt updates to these recommendations.

## Author Contributions

S.R.S.: conceived the original concept, completed literature search, led expert opinion panel survey, wrote and revised manuscript. A.D.M.: completed literature search, led expert opinion panel survey, wrote and revised manuscript. M.R.: reviewed evidence for literature support; revised manuscript. J.Y.H.C.: reviewed evidence for literature support; revised manuscript. W.M.: reviewed evidence for literature support; revised manuscript. G.R.W.: reviewed evidence for literature support; revised manuscript. L.S.R.: reviewed evidence for literature support; revised manuscript. W.R.U.: reviewed evidence for literature support; wrote and revised manuscript.

## Conflicts of Interest

The authors declare no conflicts of interest.

## Supporting information


**Data S1:** Supporting Information.

## Data Availability

The data that support the findings of this study are available from the corresponding author upon reasonable request.
