# Supplementary material for: Practice Recommendations for Genetic Testing of Ataxias
Source: Ann Clin Transl Neurol. 2025 Aug 29;12(12):2398–409. doi: 10.1002/acn3.70171 (PMC12698944; doi:10.1002/acn3.70171)
Supplement: Supplementary file 1 — Data S1: Supporting Information. [file ACN3-12-2398-s001.docx]

**SUPPLEMENTAL INFORMATION**

**SUPPLEMENTAL METHODS:**

Search Strategy

Terms related to genetic testing were limited to the title field only in order to conduct a more specific search suitable for a practice guideline.

**PubMed - 2272 on 11/6/24**

(((Friedreich*[tw] OR "adult-onset"[tw] OR Hereditary[tw] OR Inborn[tw] OR Dominant[tw] OR Recessive[tw] OR X-linked[tw] OR Xlinked[tw] OR Mitochondrial[tw] OR Cerebellar[tw] OR Spinocerebellar[tw] OR Genetic*[tw] OR inherit*[tw] OR "Fragile X*"[tw]) AND (ataxia*[tw] OR Ataxia[mesh])) OR ("Cerebellar ataxia"[mesh] OR Ataxia/genetics[mesh] OR "Fragile X-Associated Tremor"[tw] OR "Machado-Joseph Disease"[tw])) AND ("Genetic testing"[mesh] OR "Genetic Counseling"[Mesh] OR "Genetic test*"[ti] OR "exome sequenc*"[ti] OR "Molecular test*"[ti] OR "Presymptomatic test*"[ti] OR "Predictive test*"[ti] OR "Diagnostic test*"[ti] OR "Symptomatic test*"[ti] OR counsel*[tw] OR Preimplant*[ti] OR pre-implant*[ti] OR diagnos*[ti])

**Embase - 2300 on 11/6/24**

(((Friedreich*:ti,ab OR adult-onset:ti,ab OR Hereditary:ti,ab OR Inborn:ti,ab OR Dominant:ti,ab OR Recessive:ti,ab OR X-linked:ti,ab OR Xlinked:ti,ab OR Mitochondrial:ti,ab OR Cerebellar:ti,ab OR Spinocerebellar:ti,ab OR Genetic*:ti,ab OR inherit*:ti,ab OR 'Fragile X*':ti,ab) AND (ataxia*:ti,ab OR Ataxia/mj)) OR ('Cerebellar ataxia'/mj OR 'Fragile X-Associated Tremor':ti,ab OR 'Machado-Joseph Disease':ti,ab)) AND ('Genetic screening'/mj OR 'Genetic Counseling'/mj OR 'Genetic test*':ti OR 'exome sequenc*':ti OR 'Molecular test*':ti OR 'Presymptomatic test*':ti OR 'Predictive test*':ti OR 'Diagnostic test*':ti OR 'Symptomatic test*':ti OR counsel*:ti,ab OR Preimplant*:ti OR pre-implant*:ti OR diagnos*:ti)

**Scopus - 2790 on 11/6/24**

(((TITLE-ABS-KEY(Friedreich*) OR TITLE-ABS-KEY(adult-onset) OR TITLE-ABS-KEY(Hereditary) OR TITLE-ABS-KEY(Inborn) OR TITLE-ABS-KEY(Dominant) OR TITLE-ABS-KEY(Recessive) OR TITLE-ABS-KEY(X-linked) OR TITLE-ABS-KEY(Xlinked) OR TITLE-ABS-KEY(Mitochondrial) OR TITLE-ABS-KEY(Cerebellar) OR TITLE-ABS-KEY(Spinocerebellar) OR TITLE-ABS-KEY(Genetic*) OR TITLE-ABS-KEY(inherit*) OR TITLE-ABS-KEY("Fragile X*")) AND (TITLE-ABS-KEY(ataxia*) OR INDEXTERMS(Ataxia))) OR (TITLE-ABS-KEY("Fragile X-Associated Tremor") OR TITLE-ABS-KEY("Machado-Joseph Disease"))) AND (TITLE("Genetic test*") OR TITLE("exome sequenc*") OR TITLE("Molecular test*") OR TITLE("Presymptomatic test*") OR TITLE("Predictive test*") OR TITLE("Diagnostic test*") OR TITLE("Symptomatic test*") OR TITLE-ABS-KEY(counsel*) OR TITLE(Preimplant*) OR TITLE(pre-implant*) OR TITLE(diagnos*))

**Appendices**

**Appendix A: Access to Genetic Counseling Services**

Given the considerations for genetic test selection, result interpretation and implications of results for both the patient and their family members, pre and post-test counseling is essential. Optimally, in an ideal world, many of these patients would be evaluated in an Ataxia Clinic with an embedded genetic counselor, working closely with the clinical neurologist so that patients could benefit from the expertise and perspectives of both types of clinicians. This is more available in academic medical centers, but often not widely an option given workforce and fiscal limitations. We note where referrals for genetic counseling are specifically needed and content to cover in the absence of the patient being seen by a genetics provider (**Tables 2 and 3**, *Appendices B and C*).

Given the limited genetic counseling resources available depending on where patients reside, other virtual options can be considered. There are companies that independently offer virtual genetic counseling and companies that offer both genetic testing and genetic counseling. The National Ataxia Foundation (NAF), for example, has resources regarding companies offering genetic counseling and testing services (<https://www.ataxia.org/genetics/>). For some specific ataxias, free counseling and testing may be available. Of note, NAF does not endorse any one genetic testing company, and their publicly available list may not include all available options. Other online-based services are becoming available, and resources may be identified by contacting a local NAF Center of Excellence (<https://www.ataxia.org/ace>) or searching online for genetic counseling companies.

However, in certain situations, such as diagnostic testing for symptomatic patients, the neurologist should be prepared to discuss and order genetic testing as part of the diagnostic workup. In fact, a neurologist may have already established a longitudinal relationship with the individual/family members and is well equipped to address clinical questions that stem from test results. However, if there are other neurological conditions in the family, the patient may benefit from a genetics referral prior to genetic testing to determine specific test to order or post-testing to determine if additional testing is indicated given the results.

**Appendix B: Testing Options & Insurance Implications**

Genetic testing continues to evolve, both with its place in medical care and innovative sequencing technologies. With this evolution comes change in pricing and decisions of insurers to cover. Given the newness of genetic tests, there can be issues with insurance coverage given lack of evidence-based data and tests can be more costly when first introduced. An insurer may deny coverage of genetic testing in a symptomatic patient if results will not impact their medical care. This is particularly an issue for patients with Medicare insurance. We recommend that an explicit discussion be had with the patient regarding possible issues with insurance coverage of genetic testing, including potential out of pocket cost. Some labs offer benefits investigations and/or will notify patients if cost is over a set amount. Labs may offer payment plans and financial assistance/reduced costs based on income. Self-pay options generally are at a lower test cost. While our hope is that all genetic testing will be covered in the future, we do recognize that oftentimes testing is denied, at which point physician advocacy can be critical to emphasize the need for prompt and adequate testing.

**Anonymous Testing**: A patient may inquire as to whether genetic testing can be done using an alias given their concerns about privacy of results and insurance implications. This presents both counseling and logistical considerations. Some clinicians may consider such requests on a case-by-case basis. Since genetic testing is not being done under the patient’s real name, this can potentially impact their care since results are not in their medical records. Testing would need to be repeated in the patient’s name for inclusion in medical records and to obtain care based on the results.

**Sponsored Testing**: There may be sponsored genetic testing programs available for individuals with or at risk for a genetic ataxia at ‘no financial cost’ to the patient. These programs often are through partnerships between a genetic testing lab and a pharmaceutical company, patient organization, or other external donor. For the patient, this may make genetic testing feasible and eliminate the need for out-of-pocket costs or insurance billing for genetic testing. In some cases, the sponsored testing may have the same genes analyzed but could also be more or less comprehensive. Many of these programs are affiliated with a data sharing agreement that must be considered when obtaining information consent due to possible concerns associated with confidentiality and privacy.

**Young adults (ages 18-26):** Individuals at this age who are considering predictive genetic testing are generally newer to dealing with insurance issues. While the Affordable Care Act (ACA) means that the young adult can be covered under their parent(s) healthcare policy until age 26, it is important to understand this aging out limitation in coverage and the fact that results could, like for everyone, limit their ability to obtain life, disability and long-term care insurance.

**Affected individuals:** The Affordable Care Act prohibits health insurers from denying coverage or increasing premiums for pre-existing conditions in individuals who are symptomatic.

**Appendix C: Reproductive Testing**

Carrier Screening

Carrier screening is genetic testing that determines if an individual has (“carries”) a pathogenic variant on one allele for a certain disease. This is often used to identify if an individual and their reproductive partner are carriers for the same autosomal recessive condition, including some ataxias, to determine the chance of having a child with that specific condition.

Generally, carrier screening will be discussed with the patient’s primary care and/or obstetrics/gynecology provider. If the patient has a personal and or family history of ataxia, optimally the patient should see a genetic counselor/geneticist for risk assessment and selection of the appropriate genetic test. Multigene panels have different genes included and different residual risks if results are negative.

With carrier screening now being offered to all pregnant patients or those desiring to become pregnant^76^, there is expected to be increased identification of carriers of conditions associated with ataxia. These patients and reproductive partners may be seeking information about the condition of interest to make more informed pregnancy decisions and should be referred for genetic counseling^77,78^ .

Current conditions that are often included in comprehensive carrier screening panels:

- Ataxia-telangiectasia (*ATM*)
- Autosomal recessive cerebellar ataxia type 3 (*ANO10*)
- Autosomal recessive spastic ataxia of Charlevoix-Saguenay (*SACS*)
- Fragile X (*FMR1)*
- POLG-related disorders *(POLG*)

With the continued expansion of conditions included on carrier screening panels, additional conditions associated with ataxia may be identified.

Pre-implantation Genetic Testing (PGT)

Individuals with a personal and/or family history of ataxia may elect to pursue pre-implantation genetic testing (PGT), if the pathogenic variant(s) is known^79,80^ . With this testing, embryos will be created via *in vitro* fertilization and analyzed for the known pathogenic variant(s). Embryos that have inherited the ataxia can be identified and excluded from implantation.

If an individual is known to be at-risk of inheriting an ataxia but do not desire to know their own genetic status, non-disclosure PGT may be available; “may” because not all testing centers offer this option. Two methods of non-disclosure PGT include direct and indirect:

- Direct- The individual will not be informed if embryos were excluded due to a positive result of the ataxia condition or due to other reasons such as chromosomal aneuploidy. The individual’s genetic status will only be known by the PGT lab.
- Indirect- This is an exclusion-based method performed via linkage analysis where the individual’s genetic status is not known by any party; this testing requires samples from additional family members.

Prenatal Testing

Prenatal testing is available if the pathogenic variant(s) is known for the hereditary ataxia and can be performed in two ways:

- Chorionic villus sampling (CVS)- This diagnostic testing option is typically performed between 10-12 weeks gestation and can be done either transcervical or transabdominal.
- Amniocentesis- This diagnostic testing option is typically offered after 15 weeks gestation.

The availability of these prenatal diagnostic testing options and the risk of miscarriage with these procedures can vary by institution but generally is less than 1%.

**Supplemental Table S1. Evidence Summary Document**

**Section 1: Clinical Decision Making**

**Recommendation 1: All symptomatic persons should undergo a comprehensive evaluation by a neurologist prior to initiating genetic testing.**

Strength: This recommendation is supported by at least one study of level 2 evidence.

Included studies:

| **Authors and Publication Year** | **Title** | **Level of Evidence** |
| --- | --- | --- |
| van de Warrenburg et al., 2014 | EFNS/ENS Consensus on the diagnosis and management of chronic ataxias in adulthood | 2 |
| Cruz-Mariño et al., 2015 | SCA2 predictive testing in Cuba: challenging concepts and protocol evolution | 3 |
| Powell et al., 2010 | Spinocerebellar ataxia: patient and health professional perspectives on whether and how patents affect access to clinical genetic testing | 3 |
| de Silva et al., 2019 | Guidelines on the diagnosis and management of the progressive ataxias | 4 |
| Hosseinpour et al., 2024 | An overview of early-onset cerebellar ataxia: a practical guideline | 5 |
| Nance, 2003 | Genetic testing in inherited ataxias | 5 |
| Rudaks et al., 2024 | An Update on the Adult-Onset Hereditary Cerebellar Ataxias: Novel Genetic Causes and New Diagnostic Approaches | 5 |
| Tassone et al., 2023 | Insight and Recommendations for Fragile X-Premutation-Associated Conditions from the Fifth International Conference on FMR1 Premutation | 5 |

**Recommendation #2: For symptomatic persons without a known family history, non-genetic causes of ataxia should first be ruled out prior to genetic testing, unless clinical suspicion is high for a particular syndrome.**

Strength: This recommendation is supported by at least one study of level 2 evidence.

Included studies:

| **Authors and Publication Year** | **Title** | **Level of Evidence** |
| --- | --- | --- |
| van de Warrenburg et al., 2014 | EFNS/ENS Consensus on the diagnosis and management of chronic ataxias in adulthood | 2 |
| Gorcenco et al., 2024 | Clinical and genetic analyses of a Swedish patient series diagnosed with ataxia | 4 |
| Pyle et al., 2015 | Exome sequencing in undiagnosed inherited and sporadic ataxias | 4 |
| Tan & Ashizawa, 2001 | Genetic testing in spinocerebellar ataxias: defining a clinical role | 5 |

**Recommendation #3: For persons with a known family history of ataxia and a neurological evaluation consistent with that syndrome, genetic testing should be the first diagnostic test ordered.**

Strength: This recommendation is supported by at least one study of level 2 evidence.

Included studies:

| **Authors and Publication Year** | **Title** | **Level of Evidence** |
| --- | --- | --- |
| van de Warrenburg et al., 2014 | EFNS/ENS Consensus on the diagnosis and management of chronic ataxias in adulthood | 2 |
| Hess et al., 2009 | Diagnostic genetic testing for a fatal illness: The experience of patients with movement disorders | 3 |

**Recommendation 4: Ordering genetic testing should be expedited if there is high clinical suspicion of a genetic ataxia with an FDA-approved treatment.**

Strength: This recommendation is supported by at least one study of level 2 evidence.

Included studies:

| **Authors and Publication Year** | **Title** | **Level of Evidence** |
| --- | --- | --- |
| van de Warrenburg et al., 2014 | EFNS/ENS Consensus on the diagnosis and management of chronic ataxias in adulthood | 2 |

**Recommendation #5: All asymptomatic persons with a known family history of a genetic ataxia should be offered genetic counseling.**

Strength: This recommendation is supported by at least one study of level 3 evidence.

Included studies:

| **Authors and Publication Year** | **Title** | **Level of Evidence** |
| --- | --- | --- |
| Cruz-Mariño et al., 2015 | SCA2 predictive testing in Cuba: challenging concepts and protocol evolution | 3 |
| Hess et al., 2009 | Diagnostic genetic testing for a fatal illness: The experience of patients with movement disorders | 3 |
| Paneque et al., 2007 | Psychological aspects of presymptomatic diagnosis of spinocerebellar ataxia type 2 in Cuba | 3 |
| Rodrigues et al., 2012 | Presymptomatic testing for neurogenetic diseases in Brazil: assessing who seeks and who follows through with testing | 3 |
| Rolim et al., 2006 | The perceived advantages and disadvantages of presymptomatic testing for Machado-Joseph disease: development of a new self-response inventory | 3 |
| Nance et al., 2003 | Ethical Issues in Genetic Testing for Movement Disorders | 5 |

**Recommendation 6: Psychological status of every patient should be considered. While not a formal requirement prior to genetic testing, referral should be made at the discretion of the ordering provider.**

Strength: This recommendation is supported by at least one study of level 3 evidence.

Included studies:

| **Authors and Publication Year** | **Title** | **Level of Evidence** |
| --- | --- | --- |
| Cruz-Mariño et al., 2015 | SCA2 predictive testing in Cuba: challenging concepts and protocol evolution | 3 |
| Gonzalez et al., 2005 | Short-term psychological impact of predictive testing for Machado-Joseph disease: Depression and anxiety levels in individuals at risk from the Azores (Portugal) | 3 |
| Gonzalez et al., 2012 | Psychological well-being and family satisfaction levels five years after being confirmed as a carrier of the Machado-Joseph disease mutation | 3 |
| Mariotti et al., 2010 | Predictive genetic tests in neurodegenerative disorders: a methodological approach integrating psychological counseling for at-risk individuals and referring clinicians | 3 |
| Paneque et al., 2007 | Psychological aspects of presymptomatic diagnosis of spinocerebellar ataxia type 2 in Cuba | 3 |
| Paneque et al., 2007 | Psychological follow-up of presymptomatic genetic testing for spinocerebellar ataxia type 2 (SCA2) in Cuba | 3 |
| Powell et al., 2010 | Spinocerebellar ataxia: patient and health professional perspectives on whether and how patents affect access to clinical genetic testing | 3 |
| Rolim et al., 2006 | The perceived advantages and disadvantages of presymptomatic testing for Machado-Joseph disease: development of a new self-response inventory | 3 |
| Abe & Itoyama, 1997 | Psychological consequences of genetic testing for spinocerebellar ataxia in the Japanese | 4 |
| Cruz-Mariño et al., 2011 | Ethical dilemmas in genetic testing: examples from the Cuban program for predictive diagnosis of hereditary ataxias | 4 |
| McConkie-Rosell et al., 2005 | Genetic counseling for fragile x syndrome: updated recommendations of the national society of genetic counselors | 4 |
| McConkie-Rosell et al., 2007 | Recommendations from multi-disciplinary focus groups on cascade testing and genetic counseling for fragile X-associated disorders | 4 |
| White et al., 2010 | Exploration of transitional life events in individuals with Friedreich ataxia: implications for genetic counseling | 4 |

**Recommendation 7: Persons with a known personal or family history of a genetic ataxia who wish to test a pregnancy should be referred to a genetic counselor prior to or early in a pregnancy.**

Strength: This recommendation is supported by at least one study of level 2 evidence.

Included studies:

| **Authors and Publication Year** | **Title** | **Level of Evidence** |
| --- | --- | --- |
| Monaghan et al., 2013 | ACMG Standards and Guidelines for fragile X testing: a revision to the disease-specific supplements to the Standards and Guidelines for Clinical Genetics Laboratories of the American College of Medical Genetics and Genomics | 2 |
| Cahn et al., 2020 | Spinocerebellar Ataxia Patient Perceptions Regarding Reproductive Options | 3 |
| Mendes et al., 2021 | Between responsibility and desire: Accounts of reproductive decisions from those at risk for or affected by late-onset neurological diseases | 3 |
| Paneque et al., 2007 | Psychological follow-up of presymptomatic genetic testing for spinocerebellar ataxia type 2 (SCA2) in Cuba | 3 |
| Cruz-Mariño et al., 2011 | Ethical dilemmas in genetic testing: examples from the Cuban program for predictive diagnosis of hereditary ataxias | 4 |
| McConkie-Rosell et al., 2005 | Genetic counseling for fragile x syndrome: updated recommendations of the national society of genetic counselors | 4 |
| McConkie-Rosell et al., 2007 | Recommendations from multi-disciplinary focus groups on cascade testing and genetic counseling for fragile X-associated disorders | 4 |
| Abrams et al., 2012 | Newborn, carrier, and early childhood screening recommendations for fragile X | 5 |
| Berry-Kravis et al., 2007 | Fragile X-associated tremor/ataxia syndrome: clinical features, genetics, and testing guidelines | 5 |
| Dean & Ao, 2002 | Genetic prenatal and preimplantation diagnosis of trinucleotide repeat disorders | 5 |
| Gane & Abrams, 2016 | Genetic counseling for FXTAS and fragile X-associated disorders | 5 |

**Recommendation #8: Genetic testing of minors should only be offered when the minor is determined to be symptomatic by a pediatric neurologist with expertise in ataxia. Testing of minors may also be considered if there is an approved treatment or asymptomatic minors with a positive family history and predictive positive results.**

Strength: This recommendation is supported by at least one study of level 2 evidence.

Included studies:

| **Authors and Publication Year** | **Title** | **Level of Evidence** |
| --- | --- | --- |
| Sequeiros et al., 2010 | Consensus and controversies in best practices for molecular genetic testing of spinocerebellar ataxias | 2 |
| Cruz-Mariño et al., 2015 | SCA2 predictive testing in Cuba: challenging concepts and protocol evolution | 3 |
| Lowe et al., 2015 | "Both Sides of the Wheelchair": The Views of Individuals with, and Parents of Individuals with Friedreich Ataxia Regarding Pre-symptomatic Testing of Minors | 3 |
| Tassone et al., 2023 | Insight and Recommendations for Fragile X-Premutation-Associated Conditions from the Fifth International Conference on FMR1 Premutation | 5 |

**Section 2: Genetic Counseling**

**Recommendation 9: All individuals with or at risk for a suspected genetic ataxia should be offered genetic counseling.**

Strength: This recommendation is supported by at least one study of level 3 evidence.

Included studies:

| **Authors and Publication Year** | **Title** | **Level of Evidence** |
| --- | --- | --- |
| Cruz-Mariño et al., 2015 | SCA2 predictive testing in Cuba: challenging concepts and protocol evolution | 3 |
| do Nascimento Marinho et al., 2015 | Analysis of pre-test interviews in a cohort of Brazilian patients with movement disorders | 3 |
| Hess et al., 2009 | Diagnostic genetic testing for a fatal illness: The experience of patients with movement disorders | 3 |
| Mariotti et al., 2010 | Predictive genetic tests in neurodegenerative disorders: a methodological approach integrating psychological counseling for at-risk individuals and referring clinicians | 3 |
| Paneque et al., 2007 | Psychological follow-up of presymptomatic genetic testing for spinocerebellar ataxia type 2 (SCA2) in Cuba | 3 |
| McConkie-Rosell et al., 2005 | Genetic counseling for fragile x syndrome: updated recommendations of the national society of genetic counselors | 4 |
| Berry-Kravis et al., 2007 | Fragile X-associated tremor/ataxia syndrome: clinical features, genetics, and testing guidelines | 5 |

**Recommendation 10: Informed consent must be obtained before genetic testing is ordered.**

Strength: This recommendation is supported by at least one study of level 3 evidence.

Included studies:

| **Authors and Publication Year** | **Title** | **Level of Evidence** |
| --- | --- | --- |
| Rolim et al., 2006 | The perceived advantages and disadvantages of presymptomatic testing for Machado-Joseph disease: development of a new self-response inventory | 3 |
| Cruz-Mariño et al., 2011 | Ethical dilemmas in genetic testing: examples from the Cuban program for predictive diagnosis of hereditary ataxias | 4 |
| Nance et al., 2003 | Ethical Issues in Genetic Testing for Movement Disorders | 5 |

**Recommendation 11: The individual seeking genetic testing must be determined to have the capacity for decision making, unless a medical power of attorney has been appointed.**

Strength: This recommendation is supported by Expert Opinion.

**Recommendation 12: All genetic testing must be performed free of coercion.**

Strength: This recommendation is supported by at least one study of level 3 evidence.

Included studies:

| **Authors and Publication Year** | **Title** | **Level of Evidence** |
| --- | --- | --- |
| Rodrigues et al., 2012 | Presymptomatic testing for neurogenetic diseases in Brazil: assessing who seeks and who follows through with testing | 3 |

**Recommendation 13: Genetic counseling should include a discussion of the benefits, risks, and limitations of genetic testing**

Strength: This recommendation is supported by at least one study of level 3 evidence.

Included studies:

| **Authors and Publication Year** | **Title** | **Level of Evidence** |
| --- | --- | --- |
| do Nascimento Marinho et al., 2015 | Analysis of pre-test interviews in a cohort of Brazilian patients with movement disorders | 3 |
| Hess et al., 2009 | Diagnostic genetic testing for a fatal illness: The experience of patients with movement disorders | 3 |
| Rolim et al., 2006 | The perceived advantages and disadvantages of presymptomatic testing for Machado-Joseph disease: development of a new self-response inventory | 3 |
| Mendes et al., 2019 | Choosing not to know: accounts of non-engagement with pre-symptomatic testing for Machado-Joseph disease | 3 |
| Mendes et al., 2021 | Between responsibility and desire: Accounts of reproductive decisions from those at risk for or affected by late-onset neurological diseases | 3 |
| de Silva et al., 2019 | Guidelines on the diagnosis and management of the progressive ataxias | 4 |
| Schuler-Faccini et al., 2014 | Genetic counseling and presymptomatic testing programs for Machado-Joseph Disease: lessons from Brazil and Portugal | 4 |
| Nance et al., 2003 | Ethical Issues in Genetic Testing for Movement Disorders | 5 |
| Nance, 2003 | Genetic testing in inherited ataxias | 5 |

**Recommendation #14: Pre-test genetic counseling should include a discussion of current legal protections and insurance implications.**

Strength: This recommendation is supported by at least one study of level 2 evidence.

Included studies:

| **Authors and Publication Year** | **Title** | **Level of Evidence** |
| --- | --- | --- |
| Watson et al., 2024 | Cross-Sectional Analysis of Exome Sequencing Diagnosis in Patients With Neurologic Phenotypes Facing Barriers to Clinical Testing | 2 |
| Powell et al., 2010 | Spinocerebellar ataxia: patient and health professional perspectives on whether and how patents affect access to clinical genetic testing | 3 |
| Gane & Abrams, 2016 | Genetic counseling for FXTAS and fragile X-associated disorders | 5 |
| Nance, 2003 | Genetic testing in inherited ataxias | 5 |

**Recommendation 15: Post-test genetic counseling should be provided to all persons regardless of type of result.**

Strength: This recommendation is supported by at least one study of level 3 evidence.

Included studies:

| **Authors and Publication Year** | **Title** | **Level of Evidence** |
| --- | --- | --- |
| Mariotti et al., 2010 | Predictive genetic tests in neurodegenerative disorders: a methodological approach integrating psychological counseling for at-risk individuals and referring clinicians | 3 |
| Nance, 2003 | Genetic testing in inherited ataxias | 5 |
| Berry-Kravis et al., 2007 | Fragile X-associated tremor/ataxia syndrome: clinical features, genetics, and testing guidelines | 5 |

**Recommendation #16: For individuals for whom a genetic cause is identified, post-test counseling should include a discussion of carrier screening or predictive testing for at-risk family members.**

Strength: This recommendation is supported by at least one study of level 3 evidence.

Included studies:

| **Authors and Publication Year** | **Title** | **Level of Evidence** |
| --- | --- | --- |
| Archibald et al., 2009 | "It's something i need to consider": Decisions about carrier screening for fragile X syndrome in a population of non-pregnant women | 3 |
| Archibald et al., 2013 | "It's about having the choice": stakeholder perceptions of population-based genetic carrier screening for fragile X syndrome | 3 |
| Lieberman et al., 2011 | Conflicts regarding genetic counseling for fragile X syndrome screening: A survey of clinical geneticists and genetic counselors in Israel | 3 |

**Recommendation #17: Individuals undergoing genetic testing are encouraged to have a support person present, especially at result disclosure.**

Strength: This recommendation is supported by at least one study of level 3 evidence.

Included studies:

| **Authors and Publication Year** | **Title** | **Level of Evidence** |
| --- | --- | --- |
| do Nascimento Marinho et al., 2015 | Analysis of pre-test interviews in a cohort of Brazilian patients with movement disorders | 3 |

**Section 3: Genetic Testing and Laboratory Methodology**

**Recommendation 18: The initial genetic test ordered should be as comprehensive as possible, include the most ataxia-relevant genes, and reliably detect pathogenic repeat expansions.**

Strength: This recommendation is supported by at least one study of level 1 evidence.

Included studies:

| **Authors and Publication Year** | **Title** | **Level of Evidence** |
| --- | --- | --- |
| Chen et al., 2022 | Functional genomics provide key insights to improve the diagnostic yield of hereditary ataxia | 1 |
| Bonnet et al., 2023 | Optimized testing strategy for the diagnosis of GAA-FGF14 ataxia | 2 |
| Cagnoli et al., 2018 | Spinocerebellar Ataxia Tethering PCR: A Rapid Genetic Test for the Diagnosis of Spinocerebellar Ataxia Types 1, 2, 3, 6, and 7 by PCR and Capillary Electrophoresis | 2 |
| Miyatake et al., 2022 | Rapid and comprehensive diagnostic method for repeat expansion diseases using nanopore sequencing | 2 |
| Muthuswamy et al., 2016 | A Pilot Study on Assessment of Triplet Repeat Primed PCR for Fragile X Syndrome Diagnosis | 2 |
| Rajan-Babu et al., 2021 | Genome-wide sequencing as a first-tier screening test for short tandem repeat expansions | 2 |
| Sequeiros et al., 2010 | Consensus and controversies in best practices for molecular genetic testing of spinocerebellar ataxias | 2 |
| Tenorio et al, 2024 | Diagnostic Yield of NGS Tests for Hereditary Ataxia: a Systematic Review | 2 |
| van de Warrenburg et al., 2014 | EFNS/ENS Consensus on the diagnosis and management of chronic ataxias in adulthood | 2 |
| Watson et al., 2024 | Cross-Sectional Analysis of Exome Sequencing Diagnosis in Patients With Neurologic Phenotypes Facing Barriers to Clinical Testing | 2 |
| Yoon et al., 2024 | Diagnostic uplift through the implementation of short tandem repeat analysis using exome sequencing | 2 |
| Audet et al., 2023 | Integration of multi-omics technologies for molecular diagnosis in ataxia patients | 3 |
| Baviera-Muñoz et al., 2022 | Diagnostic Efficacy of Genetic Studies in a Series of Hereditary Cerebellar Ataxias in Eastern Spain | 3 |
| Boghanova-Mihaylova et al., 2021 | Inherited Cerebellar Ataxias: 5-Year Experience of the Irish National Ataxia Clinic | 3 |
| Galatolo et al., 2021 | NGS in Hereditary Ataxia: When Rare Becomes Frequent | 3 |
| Powell et al., 2010 | Spinocerebellar ataxia: patient and health professional perspectives on whether and how patents affect access to clinical genetic testing | 3 |
| Riso et al., 2021 | Application of a Clinical Workflow May Lead to Increased Diagnostic Precision in Hereditary Spastic Paraplegias and Cerebellar Ataxias: A Single Center Experience | 3 |
| da Graça et al., 2022 | Diagnostic Yield of Whole Exome Sequencing for Adults with Ataxia: a Brazilian Perspective | 4 |
| Galatolo et al., 2018 | Application of a Clinical Workflow May Lead to Increased Diagnostic Precision in Hereditary Spastic Paraplegias and Cerebellar Ataxias: A Single Center Experience | 4 |
| Gorcenco et al., 2024 | Clinical and genetic analyses of a Swedish patient series diagnosed with ataxia | 4 |
| Pyle et al., 2015 | Exome sequencing in undiagnosed inherited and sporadic ataxias | 4 |
| Sawyer et al., 2014 | Exome sequencing as a diagnostic tool for pediatric-onset ataxia | 4 |
| Chintalaphani et al., 2021 | An update on the neurological short tandem repeat expansion disorders and the emergence of long-read sequencing diagnostics | 5 |
| Dratch et al., 2024 | Genetic testing in adults with neurologic disorders: indications, approach, and clinical impacts | 5 |
| Gorcenco et al., 2020 | New generation genetic testing entering the clinic | 5 |
| Rudaks et al., 2024 | An Update on the Adult-Onset Hereditary Cerebellar Ataxias: Novel Genetic Causes and New Diagnostic Approaches | 5 |
| Sandford & Burmeister, 2014 | Genes and genetic testing in hereditary ataxias | 5 |
| Novis et al., 2020 | Evidence and practices of the use of next generation sequencing in patients with undiagnosed autosomal dominant cerebellar ataxias: A review | 5 |
| Wallace et al., 2018 | Molecular genetic testing for hereditary ataxia: What every neurologist should know | 5 |

**Recommendation 19: Re-analysis of previously negative genomic testing should be considered after a period of at least 2 years or a change in symptomology.**

Strength: This recommendation is supported by at least one study of level 4 evidence.

Included studies:

| **Authors and Publication Year** | **Title** | **Level of Evidence** |
| --- | --- | --- |
| Gorcenco et al., 2024 | Clinical and genetic analyses of a Swedish patient series diagnosed with ataxia | 4 |

**Recommendation 20: DNA banking should be offered to all symptomatic individuals fora known or suspected genetic ataxia who either have negative results or decline testing.**

Strength: This recommendation is supported by Expert Opinion.

**Supplemental Table S2. Implications of the types of genetic test results**

| **Result** | **Implications** |
| --- | --- |
| Positive | A confirmatory gene change/mutation (pathogenic variant) known to cause a genetic ataxia has care implications for both the patient and family members. If the individual has not seen a neurologist with expertise in hereditary ataxia, then this should be arranged. Once care has been established, the individual should continue regular follow-up with the neurologist for ongoing medical management. Family members at risk for developing the identified genetic ataxia should be triaged into either diagnostic or predictive testing depending on whether symptomatic or asymptomatic and follow above recommendations. |
| Variant of Uncertain Significance | Given the newness of these genetic tests and the number of genes analyzed, it is not unusual to have a single or multiple variant(s) of uncertain significance (VUS) identified. A VUS means there is not enough data evidence to determine whether pathogenic or benign. Most of the time (80%+), a VUS is reclassified as benign^70^.  When there is a VUS, sometimes the lab will note in the report the option to test additional family members at no cost in an effort for variant resolution. If not offered and the clinician has strong suspicion that the VUS could be pathogenic, it may be possible to make a case for the lab to do so. Determining whether a VUS is inherited, or *de novo* may not be feasible if key family members’ samples are unavailable (e.g. parents, additional affected family members). Testing relevant family members’ samples may yield additional evidence as to whether a variant is trending towards benign or pathogenic but may not be sufficient to reclassify given the established criteria for variant interpretation^71^.  The challenging concept to convey to patients is that while a VUS may have been identified in a gene associated with an ataxia, it may in fact not be the cause. It should be a shared responsibility for labs to contact the ordering provider about a VUS reinterpretation, for the ordering provider to contact the lab for an update periodically when the patient is seen and for the patient to contact the ordering provider to determine whether a VUS has been reinterpreted and/or if additional genetic testing needs to be considered^72,73^. |
| Intermediate | For many genetic ataxias caused by a repeat expansion, there is a range of repeat sizes that may not be classified as either pathogenic/disease-causing or benign but instead as “intermediate.” This has also been referred to as “gray zone” results in some instances^74^. These ranges often are based on factors such as likelihood of expansion in future generations, phenotypic presentations, and/or molecular mechanisms. Often these results must be interpreted based on the consistency of the patient’s phenotypic presentation with the condition of interest. As additional research becomes available for each respective genetic ataxia, these intermediate ranges may change resulting in a patient’s result subsequently being reported as positive or negative.  For some conditions, there may be clinical heterogeneity associated with an intermediate repeat size (e.g. *ATXN2* intermediate repeat associated with increased risk of ALS)^75^ . Additionally, given that intermediate repeats may expand into pathogenic expansions in the next generation, this could impact living children and future reproductive decision making. |
| Negative | Even with strong clinical suspicion, genetic tests may often return ‘negative’ (no change found) which prompts consideration of a few limitations of genetic testing:   1. We do not know every gene implicated in every ataxia. 2. We do not know every change in the genes that we *do* know lead to ataxia 3. Additional genetic testing may be a consideration (e.g. whole genome sequencing with long-range orthogonal confirmation). There are cost and yield limitations.   If a pathogenic variant is known in the family and an asymptomatic patient tests negative, this is considered a true negative. If a pathogenic variant is not known in the family and there is no affected individual to test, a negative result reduces the risk, and residual risk will depend on the ataxia and test sensitivity. |
